# Past and Present Teachings in the Use of Gold Foil

**Published:** 1888-08

**Authors:** James Truman


### PAST AND PRESENT TEACHINGS IN THE USE OF GOLD FOIL.

BY PROF. JAMES TRUMAN.

READ BEFORE THE PENNSYLVANIA STATE SOCIETY, JUNE 6, 1888.

The peculiar characteristics of gold foil are now so well understood that it would be a work of supererogation to attempt to instruct any body of this kind, and I do not propose to attempt it. Yet there are points that must be alluded to in order that the subject may be made clear.

If I were to define soft foil, I would say it is that form of foil that has a minimum amount of cohesiveness, and that this cohesiveness cannot be increased by heat. The definition I would give to cohesive foil would be : That form of gold which, freshly prepared, will cohere without force, and at all times may have that quality increased in degree by heat. Each of these forms find their best examples in the open market. I know of but two strictly non-cohesive foils. There may be others, but I am not familiar with them. There are many that simulate strictly non-cohesive foils, and probably answer all the purposes served by them ; but they do not answer to the definition as I have given it. It was the strictly soft foil with which the profession began its work and for, at least, a half a century, prior to 1855, did with it all the work that was done in the stopping of cavities. It was at this period, 1855, that Dr. Arthur introduced the cohesive variety of foil, and thus the century has been nearly equally divided on the two prominent forms of foil, with the result that the profession has less positiveness in its opinions to-day on the subject, of the proper methods of filling teeth, than at any time since the beginning of the century. This is all the more remarkable when it is considered that the last thirty years have developed the best operators and the most intelligent system of dentistry the world has ever seen. What is equally remarkable is, that the profession, as such, has quietly settled down and written *finished* over the temple of their construction and look askant at any one who suggests the possibility that no temple of human construction can be regarded as ever complete, but must be continually subject to repairs and remodelling. This probably arises from what appears to be a law of mind, that everything seems to move in circles and in its revolution reaches ebb and flow, low tide of interest in any special work, and then the full flood. This has been most markedly manifest in many phases of dentistry, notably in that of the mechanical branch. Forty years ago this was at the flood. Nothing was thought of but the proper way to make plates. Then in the process of revolution came the flood tide for fillings and the ebb in mechanics. Now it is flood with scientific dentistry and decidedly ebb with fillings, and prosthetic dentistry is surging to the surface and high water again with its bridge work and crowns. We have nearly reached solid rock with stoppings, and, it seems to me, if there is not more attention given to this subject in journals and conventions, we are doomed to a retrogression in the filling of teeth if, indeed, the time does not come when it will be classed with Phillips' lost arts. There is a law always operating that stagnation means death. It is true of mental development and equally true of all forms of active human effort. That the filling of teeth has received no attention in recent years can only be accounted for by the fact that interest has been measurably lost, and it behooves us to inquire whether this lack of interest does not mean not only death to skill in the handling of gold, but that it points unerringly to the near future when thoroughly good operators will be as scarce as they were in 1840, when the ten digits would more than compass the number in this country. Some will doubtless regard this as the pessimistic thought of one who has measurably left the current of active interest in this direction, and sees only through a glass darkly. But while this is to some extent true it is impossible to avoid the reflection that effects are always, or at least, should be traceable to a cause, and if the cause contains within itself the germs of imperfection, it requires no prophet to outline future results.

The operations with soft foil were based solely on certain principles, not difficult to master in theory, but exceedingly difficult to put in practice. It was not surprising, therefore, that while all, perhaps, could theoretically explain what was meant by a good soft foil filling, there were but few equal to the details as then understood. These details began primarily with the formation of the cavity, and the prognosis in every case must take in the density, thickness of walls, and position of every tooth. No general law of formation was or could be formulated. Each cavity was a law unto itself, and hence the teachers of forty years ago in operative dentistry were, perforce, compelled to train the minds of the students, not in general principles to be worked out in detail, but in details to the entire negation of nearly all general principles of procedure. When Prof. Elisha Townsend, the most finished operator of his day, found it necessary to spend nearly the entire session on cavities of all forms and the possible modes of preparation, he simply adopted the best and only reasonable mode of teaching, for each had to be studied as a separate entity. In this way many of us were trained, and when Harris explained his mode in a similar way in his "Principles and Practice of Dental Surgery," the dental world regarded the work of filling teeth as practically completed, and that no better forms of instruction could be devised. But even with this character of gold new forms of more convenient shape were prepared at a later day; but while these aided in the operation the general modes of formation of cavities remained the same. While the processes in use with non-cohesive foil were very similar, that of Prof. Townsend came nearer modern cohesive foil methods, inasmuch as he inserted his gold in small pieces, and practically built up the filling lamina by lamina. But this was not the general method. It was regarded by the average operator as slow and tedious, and only adapted to one who could charge fabulous prices from the millionaires of the period.

The method then in vogue might be appropriately called "stuffing" the cavity, a method at once uncertain and often dangerous to the tooth from the lateral force, necessarily exerted in the condensation of the filling. Notwithstanding this and other difficulties it is not surprising that results were often more satisfactory than present modes, for the perfect adaptation of the soft foil to the walls prevented imbibition of fluids at the most vulnerable point, and the enamel was not "crazed" by the blows — a thousand or more to the minute — of electric and mechanical mallets.

But in whatever light we may view the teaching and the practice of this period, it must be regarded at once imperfect and wholly without system. Teaching to be effective must first be based on a method capable of being reduced to law, and then the law should be formulated and closely adhered to. No positive law could be formulated with soft gold, and hence students were a law unto themselves. That the influence of this method has reached our day no one can deny; indeed its power is beginning to be so strongly felt that it threatens to overwhelm the work of the last quarter of a century, if it does not eventually sink all the science of filling teeth evolved during that period.

The second period was, as I before remarked, ushered in by Dr. Arthur. This has been a remarkable epoch in this special work. To some of us who recall the beginnings, the slow approaches made to any satisfactory use of cohesive foil, the tedious upbuilding of means and processes, the manufacture of appliances, all, as we think them over, savor of the marvellous; but they are all pre-eminently in harmony with the wonderful creations of this teeming century. The introducer of cohesive foil never anticipated the remarkable results lying dormant in the "sticky foil" he presented as a possible aid in dental operations, and the dentist of the time, equally oblivious, struggled with its clogging, and manfully tried to evolve something out of its intractable crystals. This was cohesive foil in 1855. It was not long, however, before chaos yielded to system, but this was the result of the working of many minds and many nimble fingers. Hours, days, weeks and years of thought were devoted to perfecting this process. First came the necessity for a proper form of cavity. Then it was manifest that something more than the old anchorages, simply places scooped out in the dentine, were necessary. This cohesive gold must be started right. It must be solid from the foundation, and this must not be left to chance. This led to the formation of bolt-like holes drilled into the tissue, which, when filled, served not only as an anchorage, but as a point to build from. This form of anchorage was, I believe, first taught by myself; at least it was original in my teaching, and the first published paper describing it was inserted in the Dental Times, July, 1865. It met with positive opposition as well as favor; but I have never been able to discover any serious objection to it to this day. Gradually the system became more and more perfectly developed, until the introduction of mallet force, which effected a radical change. This was still further increased by the multiplication of force in mechanical mallets, and then came the gradual and systematic perfection of this mode of condensation. From the introduction of cohesive foil until the final consummation which brings us down nearly to the present period, there has been a system of filling perfected with it, without perceptible flaw in its arrangement, capable of meeting all emergencies, adapted to the frailest of teeth, and capable of being taught to the dullest comprehension. The result has been better operators, and work that has stood the test of attrition and time more satisfactorily than the old mode. Whether this be acceded to or not, I claim for it that it is the only system worthy to be dignified by that title. It has defects and one of these I have already hinted at and more elaborately stated in my paper on "Rotation as a Condensing Force," read before the Odontological Society of this city. The mode of using this foil is too familiar to you to risk your patience by repeating. My effort now is to arrive at some definite conclusions in regard to these two methods and their relative value as systems of practice.

The cohesive foil method, perfect as it is as a system of filling, has met with opposition; indeed that opposition began with its introduction and has grown in strength, until at this time dentists are divided into many schools of practice. The first positive blow was struck by the, so called, New Departurist, and whatever may be said derogatory to their methods and modes of procedure, this much must be accorded them that they led the way in a revolution that now threatens to sap the very foundations of gold filling as it has heretofore been understood, and promises to land us in a chaos of conflicting ideas and practices from which it may take years to recover.

The two positive schools, in the use of gold, are composed of those who regard the first form, soft foil, as the only true method, and those who still adhere to the cohesive with its perfect system.

While I have nothing but admiration for the latter, as a system, I am not wholly in love with it as a method of preservation of tooth structure. The best years of my life were given to its perfection, and it may be said without egotism, that as professor of operative dentistry in the Pennsylvania College of Dental Surgery, I did my part to make it a positive practice, and helped to send forth an army of young men well equipped for service, and the work has made its mark on this generation. While all this is felt there are some weak points in the system that have driven many men to the adoption of a middle course, in the use of a combination of soft and cohesive foils. The use of this combination is based, 1) On rapidity of execution. 2) Adaptation of soft foil to the walls. 3) A solidity which, if not equal to cohesive alone, meets all the requirements of a good filling. When this mode is confined within certain limits, that is, as a lining to the walls and cervical border, no serious objection can be urged; indeed it is a question whether it is not the best mode of filling, as it is the quickest. When rotation (Herbst's method) is applied to the adaptation of the soft foil, no better results can be obtained, I think, by the mechanical mallet, although it gives greater density. This, to my mind, is the most practical mode of filling teeth. I do not say that it is the most perfect mode, if solidity be the test of perfection ; but in the saving of time, money to our patients, and with satisfactory results, it must be regarded, while not new, certainly the most available and practical method.

But we have other uses for fillings beside that of satisfying our strength and our patients' pockets. The filling of teeth has to be taught. It is a prominent subject in the curriculum of our colleges. It is made, in connection with mechanical dentistry, the basis of the superstructure which has been in course of construction from the time of the fathers, and will continue unfinished through many generations. Is then the first method, which I have attempted to generalize, or the last the proper mode to train either students in our offices or in our colleges? I have no hesitation in answering this, as regards both, in the negative. We should, if I view the matter correctly, start with the simplest idea, and carry it to its complications. We should begin with the conception of, perhaps, the lowest form of mechanical force—the bolt—and aspire to complexity when the mind has been trained through regular gradations. The student of all periods is necessarily a one idea man, and he must be approached upon that principle, gradually enlarging thought, and by thus enlarging we assist him to grasp more easily other ideas and modes of procedure. This will enable him to master without difficulty all forms of gold work, whether it has entered into his teaching or not. This must be regarded as a truism; but it is lost sight of in a measurable degree in the effort to jump the foundation. We cannot build houses in the air, and we cannot make dentists unless we train their hands and place in their minds systematized thought. The adoption of cohesive gold in our college clinics forms the best and only means of thoroughly instructing students. Any one who has mastered the art of manipulating cohesive foil cannot fail in the use of soft foil, if in after years it becomes advisable to adopt it to the exclusion of cohesive foil. We therefore dwell upon this point in our lectures and clinics. The transition from cohesive gold to soft and cohesive gold is easy and natural, the former having been properly acquired; but to change from soft to cohesive foil is almost an impossibility, and requires a moral force and manual dexterity not given to all men.

Hence in summing up the thoughts of this paper, I may say that the conclusion arrived at is, that if we are to maintain the present supremacy of being the best gold operators of the world, we must adhere, educationally, to the system of cohesive gold, with its anchorages and perfect welding of parts. If, on the other hand, we desire subsequently to change to a mode less laborious, more capable of giving good pecuniary returns, which will lessen strain on the patient, and, possibly, may be better as a preservative of tooth structure, we must adopt soft foil at the margins, and cohesive to resist the force of attrition in mastication.

